# Disorders of the Cerebral Circulation

**Published:** 1848-05

**Authors:** 


### DISORDERS OF THE CEREBRAL CIRCULATION.

The same publishers have issued the work of Dr. Burrows on *Diseases of the Cerebral Circulation*, a copy of which is before us. Like the treatise of Solly on a kindred subject, it is a well looking volume, and, without pretending to be a complete monograph on the brain, gives an exceedingly satisfactory account of all the topics discussed. It ought to be in the library of every physician, both for his own sake, and the sake of his students.

